# 白细胞介素-6相关抗体治疗多发性骨髓瘤的研究进展

**DOI:** 10.3760/cma.j.issn.0253-2727.2021.06.017

**Published:** 2021-06

**Authors:** 亮 任, 澎 刘

**Affiliations:** 复旦大学附属中山医院血液科，上海 200032 Department of Hematology, Zhongshan Hospital, Fudan University, Shanghai 200032, China

多发性骨髓瘤（MM）是血液系统中第二大常见肿瘤，其病理特征是骨髓中出现异常增殖的克隆性浆细胞。

白细胞介素-6（IL-6）作为一种细胞因子，在浆细胞的分化、成熟中扮演重要角色。IL-6通过作用于IL-6受体（IL-6R）参与细胞内JAK1/STAT3信号通路传导，进而影响MM细胞的生存；同时，IL-6可以上调B细胞淋巴瘤蛋白-2（Bcl-2）、髓样细胞白血病蛋白-1（Mcl-1）等抗凋亡蛋白阻止MM细胞凋亡[1]。研究表明，与早期患者相比，晚期MM患者循环中IL-6的浓度更高，且与不良预后显著相关[2]。通过抑制IL-6及其受体下游通路可能会使MM患者受益。本文对IL-6相关药物参与MM临床治疗的研究进展作一综述。

一、IL-6相关抗体在骨髓瘤联合治疗中的作用

IL-6对骨髓瘤细胞的增殖、生存有至关重要的作用，研究发现，抗IL-6抗体在体内、体外均能抑制骨髓瘤细胞的增殖。IL-6单克隆抗体在临床前模型中展现了良好的药物协同作用，增强了包括硼替佐米、美法仑、地塞米松等在内的治疗MM药物的疗效[3]。

1. Siltuximab：复发难治MM患者体内IL-6浓度更高。Siltuximab（一种人鼠嵌合抗IL-6单克隆抗体）与人体内的IL-6有极高的亲和力。在采用BS方案（硼替佐米+Siltuximab）治疗复发难治MM患者的临床试验中，同硼替佐米单药方案相比，尽管BS方案组患者客观缓解率（ORR）（55％对47％）和完全缓解（CR）率（11％对7％）更高，但患者并未在无进展生存（PFS）和总生存（OS）上显著获益[2]。而在选用Sd方案（Siltuximab+地塞米松）时，临床评估ORR为23％，无患者获得CR[4]。Suzuki等[5]将硼替佐米加入Sd方案，疗效评估2例（22％）患者达到了CR，高于两药方案（BS、Sd方案）的CR率，提示三药方案可以使患者明显获益。

对于初治MM患者，一项临床试验分别采用VMPS方案（硼替佐米+美法仑+泼尼松+Siltuximab）和VMP方案（硼替佐米+美法仑+泼尼松），结果显示ORR分别为88％和80％，非常好的部分缓解（VGPR）率分别为71％和51％（*P*＝0.0382），但两组PFS、OS的差异无统计学意义[6]。Shah等[7]应用VRD方案（硼替佐米+来那度胺+地塞米松）联合Siltuximab治疗初治MM患者，结果显示ORR为90.9％，VGPR率为45.5％，其中VGPR率较VRD方案略高[8]。

大多数MM患者会经历意义未明的单克隆免疫球蛋白增多症（MGUS）、冒烟型骨髓瘤（SMM）前驱状态，对MGUS、SMM早期干预可能会延缓疾病进展甚至抑制其向MM转化。一项临床试验对MGUS患者早期应用Siltuximab，其中3例患者M蛋白降低50％以上，9例患者M蛋白较基线下降25％[9]。Brighton等[10]开展的临床试验则纳入了高风险SMM患者，结果表明，干预组和对照组的1年PFS率分别为84.5％和74.4％，干预组疾病进展发生率较对照组低（32.6％对42.9％），Siltuximab组中位PFS时间未达到（*HR*＝0.50，*P*＝0.060）。

从安全性上评价，参与临床试验的患者均能耐受Siltuximab，没有观察到剂量限制毒性（DLT），但试验组发生了更多的血液系统相关不良事件，感染率也更高[2],[5]–[6]。此外，Chari等[11]还报道1例患者出现了体重快速增加不良事件。

2. Tocilizumab（托珠单抗）：也有IL-6R单克隆抗体用于MM患者治疗的报道。早在1997年就有研究报道应用一种人源化抗IL-6R单克隆抗体托珠单抗（Tocilizumab）治疗难治MM患者，临床监测显示患者体内的M蛋白水平得到了控制，且一定程度上改善了患者的全身水肿和发热症状[12]。也有报道显示托珠单抗用于治疗并发新型冠状病毒肺炎（COVID-19）的MM患者，控制患者体内的炎症因子风暴，并获得了良好的治疗效果，但是对MM的影响仍有待研究[13]。研究者给予8 mg/kg剂量的托珠单抗治疗伴SMM的类风湿关节炎患者，疗效评估显示，在改善关节症状的同时，可以长期稳定患者体内的M蛋白，表明托珠单抗可以在一定程度上延缓疾病进展，为在高危MGUS患者中应用托珠单抗进行早期干预提供了一些思路[14]。托珠单抗在患者体内耐受性良好，且没有显著不良事件发生。

NRI是通过托珠单抗基因改造获得的单链形式的IL-6R单克隆抗体，在降低免疫原性的同时，其成本也更经济。研究者将其注射到小鼠体内，发现可以显著抑制骨髓瘤细胞的增殖；另外一种IL-6R的超级拮抗剂Sant7可以完全抑制IL-6/IL-6R异构体的形成，从而阻断IL-6信号通路的传导，与地塞米松、全反式维甲酸联合应用在体内、体外均展现了抑制骨髓瘤细胞增殖和促进凋亡的效应[12]。

3. Ruxolitinib（芦可替尼）：芦可替尼是一种作用于JAK1/JAK2的酪氨酸激酶抑制剂，通过抑制STAT3的磷酸化阻断承接IL-6的JAK1/SATA3信号通路，进而发挥抗肿瘤作用。体内、体外实验表明芦可替尼同地塞米松、来那度胺、硼替佐米等联合应用可以发挥协同抗骨髓瘤细胞的效应[15]–[17]。临床前研究发现芦可替尼可以上调骨髓瘤细胞表面的CD38表达，从而增强达雷妥尤单抗对骨髓瘤细胞的毒性[18]。Chen等[19]的研究还表明芦可替尼可以改善骨髓瘤细胞对来那度胺的耐药性。因此Berenson等[20]对复发难治MM患者开展了一项Ⅰ期临床试验，入组标准是对包括蛋白酶体抑制剂和免疫调节剂的至少两种方案或疗法耐药。结果显示，纳入了28例患者，ORR为38％（CR率为4％，VGPR率为8％，PR率为26％），12例临床评估有效的患者均为来那度胺耐药。最常见的不良事件是贫血（57％的患者发生），36％的患者发生了严重的不良事件，包括败血症（11％）、肺炎（11％）。此外，目前尚在开展一项临床试验以评估CRD方案（卡非佐米+芦可替尼+地塞米松）对卡非佐米耐药患者的临床疗效（NCT03773107）。芦可替尼有望为复发难治MM患者提供更多治疗选择。

二、IL-6相关抗体在MM免疫疗法中的应用

近年来肿瘤免疫疗法的兴起为肿瘤治疗开辟了新局面。尤其是免疫检查点抑制剂和嵌合抗原受体T细胞（CAR-T）疗法在临床应用中取得了良好疗效。IL-6广泛参与肿瘤调控的关键通路，在肿瘤免疫疗法中也发挥了重要作用。

1. PD-1/PD-L1：PD-1/PD-L1免疫检查点抑制剂通过阻止肿瘤细胞的免疫逃逸增强人体免疫系统的抗肿瘤能力。Tamura等[21]的研究发现，IL-6可促进骨髓瘤细胞表面PD-L1表达。研究者将人骨髓瘤细胞（HMCL）和人间质细胞（HS-5）在体外共培养，发现IL-6上调了骨髓瘤细胞表面的PD-L1，这一效应呈显著的剂量相关性，通过添加IL-6单克隆抗体可在一定程度上中和IL-6的诱导效应。Tsukamoto等[22]的研究进一步证实IL-6单克隆抗体和PD-L1单克隆抗体联合应用可以发挥协同抗肿瘤作用。此外有研究表明，骨髓基质细胞分泌的IL-6参与了调节性T细胞（Treg）的转化。研究者发现，将MM细胞和骨髓基质细胞共同培养时，骨髓基质细胞分泌的IL-6可以将外来的Treg分化为分泌IL-17的T细胞，中和部分Treg的免疫抑制效应，这一现象在动物实验中得到了进一步验证[23]。因此，通过靶向IL-6调节体内免疫相关分子的抗肿瘤效应，可以使免疫疗法的效果最大化。

目前相关药物已被批准用于复发难治霍奇金淋巴瘤（cHL）和原发纵隔大B细胞淋巴瘤（PMBCL）的治疗。骨髓瘤细胞表面的PD-L1表达明显高于正常浆细胞，PD-1单药和联合方案治疗MM的临床试验正在进行中。

2. CAR-T细胞：IL-6是由活化免疫细胞分泌的细胞因子，主要来源于单核细胞，在CAR-T细胞疗法的临床应用中也发挥了重要的辅助作用。CAR-T细胞输注至患者体内后能否有效分化、扩增是患者产生应答的关键。有学者采用流式细胞术测定外周血中CAR-T细胞的数量，发现其与外周血中的IL-6存在显著相关性（*r*＝0.86，*P*＝0.01）[24]。Fraietta等[25]分析了治疗无效和有效患者体内的CAR-T细胞，发现后者体内高功能CAR-T细胞有更多IL-6/STAT3基因表达，血清IL-6水平也更高（*P*<0.05），且与体内CAR-T细胞扩增的峰值有直接相关性；使用IL-6/STAT3阻断剂可抑制CART细胞增殖，表明IL-6/STAT3信号通路对于CAR-T细胞在体内的增殖、长期存活至关重要。当前体外制备CAR-T细胞时主要添加IL-2、IL-7、IL-15等以调节T细胞的表型和质量，未来有望进一步通过添加IL-6获得更好的细胞分化及增殖潜能更高的效应T细胞，提升CAR-T细胞疗法的效果。

制约患者通过CAR-T细胞疗法获益的另一关键因素是其主要不良事件——细胞因子释放综合征（CRS），患者的主要表现是高热，但可引发危及生命的并发症：包括心功能不全、肾功能衰竭、呼吸窘迫综合征等。现有证据认为IL-6是介导CRS毒性的中枢细胞因子。Zhao等[26]进行的靶向B细胞成熟抗原（BCMA）CAR-T细胞治疗MM的临床试验表明，患者CRS的严重程度与血清中IL-6的峰值呈正相关。Cohen等[27]也证实高级别CRS患者血清中IL-6水平也更高（*P*＝0.014）。临床试验表明托珠单抗可以很好地控制CRS相关症状，其在2017年被FDA批准用于治疗CAR-T细胞疗法引起的严重、危及生命的CRS。托珠单抗对体内CAR-T细胞扩增和抗肿瘤效应的影响尚存在争议，尚有临床实践表明托珠单抗可能会增加患者神经毒性的发生率和严重程度[28]。因此，托珠单抗治疗CRS的安全性仍有待评估。异基因CAR-T细胞同自体CAR-T细胞疗法相比，在T细胞抗肿瘤潜力等方面有一定的优势，但移植物抗宿主病（GVHD）是影响患者临床获益的主要不良事件。研究发现供者CD4^+^ CAR-T细胞来源的IL-6是GVHD发生的主要驱动因子[29]，可以在患者输注CAR-T细胞前、后适度抑制IL-6/IL-6R预防GVHD。

三、IL-6单克隆抗体在造血干细胞移植中的应用

造血干细胞移植是治疗MM的重要手段，最新发布的《中国多发性骨髓瘤诊治指南（2020）》中强调了自体造血干细胞移植（auto-HSCT）在MM临床治疗中的地位[30]。研究表明，IL-6R的基因拷贝数与MM患者auto-HSCT后长期生存有显著相关性。Kim等[31]发现MM细胞IL-6R基因拷贝数更低的患者auto-HSCT后5年OS率较拷贝数更高的患者显著增加（78％对44％，*P*＝0.024）。也有研究者尝试将IL-6单克隆抗体加入MM移植前预处理方案中，从而提升auto-HSCT的疗效。Moreau等[32]采用BE-8（一种鼠抗IL-6抗体）、地塞米松和美法仑联合的预处理方案，同美法仑单药方案相比，患者在auto-HSCT后获得了更高的CR率（37.5％对11.1％，*P*＝0.058），且患者对BE-8耐受良好，无显著增加的不良事件。但在IFM99-04试验中，166例实施序贯auto-HSCT的患者随机接受地塞米松+美法仑和地塞米松+美法仑+BE-8二次移植预处理方案，虽然后者CR率更高，但差异无统计学意义（30.6％对34.6％，*P*＝0.620），患者的OS也未获益[33]。此外，在auto-HSCT前后使用IL-6单抗（Siltuximab）改善移植后不良反应、提升患者生活质量的临床试验也正在进行中（NCT03315026）。

四、结语

IL-6对体外MM细胞增殖、生存起着至关重要的作用，在体内阻断IL-6可以抑制骨髓瘤细胞的增殖。在以IL-6和相关通路为靶点药物的临床应用中，IL-6单克隆抗体在体内展现了良好的IL-6抑制能力，表明了一定程度的MM细胞增殖抑制作用，但是乏善可陈的临床疗效限制了其适应证的拓展。在免疫治疗时代，无论是对于CAR-T细胞疗法还是免疫相关药物，IL-6R单克隆抗体能够抑制严重的CRS。IL-6在MM细胞生存中的关键地位仍然吸引研究者探索以其为靶点的治疗手段。
